# Transcriptome Analysis in the Head Kidney of Rainbow Trout (*Oncorhynchus mykiss*) Immunized with a Combined Vaccine of Formalin-Inactivated *Aeromonas salmonicida* and *Vibrio anguillarum*

**DOI:** 10.3390/vaccines9111234

**Published:** 2021-10-22

**Authors:** Jongwon Lim, Suhee Hong

**Affiliations:** Department of Marine Biotechnology, Gangneung-Wonju National University, Gangneung 25457, Korea; myobong@gwnu.ac.kr

**Keywords:** vaccine, *A. salmonicida*, *V. anguillarum*, transcriptome, immune

## Abstract

This study aimed to identify the molecular mechanisms regulated by a combined vaccine against *Aeromonas salmonicida* and *Vibrio anguillarum* (O1 serotype). These bacteria cause furunculosis and vibriosis, respectively, and are associated with a high mortality in rainbow trout in Korea. The vaccine upregulated gene expression of TCRα, T-bet, sIgM, and mIgM, markers of an activated adaptive immune response. On days 1, 3, and 5, transcriptome analysis revealed 862 (430 up- and 432 downregulated), 492 (204 up- and 288 downregulated), and 741 (270 up- and 471 downregulated) differentially expressed genes (DEGs), respectively. Gene ontology (GO) enrichment analysis identified 377 (108 MF, 132 CC, 137 BP), 302 (60 MF, 180 CC, 62 BP), and 314 (115 MF, 129 CC, 70 BP) GOs at days 1, 3, and 5, respectively. Kyoto Encyclopedia of Genetic and Genomic enrichment analysis identified eight immune system-related pathways like cytokine-cytokine receptor interaction, NF-kappaB signaling pathway, TNF signaling pathway, NOD-like receptor signaling pathway, cytosolic DNA sensing pathway, cell adhesion molecule, complement and coagulation cascade, and antigen processing and presentation. In the analysis of the protein–protein interaction of immune-related DEGs, a total of 59, 21, and 21 interactional relationships were identified at days 1, 3, and 5, respectively, with TNF having the highest centrality at all three time points.

## 1. Introduction

Rainbow trout (*Oncorhynchus mykiss*) is a cold-water fish popular for aquaculture worldwide due to its rapid growth rate and fertility [1]. In Korea, it is a major inland aquaculture species after eel (*Anguilliformes*), golden apple snail (*Pomacea canaliculata*), catfish (*Silurus asotus*), and crucian carp (*Carassius carassius*) [2]. *Aeromonas salmonicida* and *Vibrio anguillarum* are gram-negative bacteria associated with major diseases in Salmonids, leading to high mortality and morbidity [3,4]. *A. salmonicida* is the causative agent of furunculosis with symptoms of muscle ulceration, hemorrhagic sepsis, and death [4]. *V. anguillarum* causes vibriosis in Salmonids and other fish species and is one of the most frequent aquatic diseases worldwide [5]. In Korea, only serotype 1 of *V. anguillarum* has been isolated so far [6].

Several studies have been conducted to investigate the immune system’s involvement in response to bacterial infection in rainbow trout. These studies have greatly improved our understanding of the host’s immune defense mechanisms against bacterial infection. For instance, it has been demonstrated that antigen processing and presentation, phagocytosis, and cell adhesion molecules (CAMs) were activated in both the spleen and head kidney of *V. anguillarum*-infected sea bream (*Pagrus major*) [7]. Indeed, recent transcriptome analyses help to understand changes in host genes during host–pathogen interactions. For example, transcriptomic analysis was performed to study the protective response to *A. salmonicida* infection in rainbow trout fed with β-glucan [8]. In addition, transcriptome profiling was performed upon infection with *A. salmonicida* and *V. anguillarum* in rainbow trout [9]. However, most of the transcriptomics studies on *A. salmonicida* and *V. anguillarum* have focused on bacterial pathogenicity and the host’s immune mechanisms after administration of live pathogens to fish.

*A. salmonicida* and *V. anguillarum* infections have been prevented using commercialized vaccines. An inactivated vaccine against *A. salmonicida* was developed in 1942 for oral use [10], and a vaccine against vibriosis was first published in 1964 by Hayashi et al. [11]. Since then, vaccines for both pathogens have been developed in various forms, including subunits and polyantigens, as well as in the form of DNA vaccines [12,13,14,15,16]. However, there is no commercialized vaccine for *A. salmonicida* and *V. anguillarum* in Korea.

Many fish vaccines are multivalent vaccines since they are beneficial for reducing handling costs. For example, *Yersinia ruckeri* antigens based on Danish strains were added to *A. salmonicida* and *V. anguillarum* vaccines to evaluate their protective efficacy [17]. In turbot (*Scophthalmus maximus*), a combined vaccine of attenuated *V. anguillarum* and *Edwardsiella piscicida* was found to induce a strong immune response [18]. In addition, the efficacy of multivalent formalin-killed cell vaccines against *V. alginolyticus* and *V. parahaemolyticus* was evaluated and found to be effective in Gilthead bream (*Sparus aurata*) [19]. A combined vaccine of inactivated *Yersinia ruckeri*, *A. salmonicida,* and *V. anguillarum* was observed to upregulate IL-10, IgD, and major histocompatibility complex (MHC) II gene expression. However, the sampling time point was too late to observe significant changes in the expression of other genes [17].

Microarray analysis revealed that administration of an attenuated *A. salmonicida* or *V. anguillarum* vaccine contributed to immune protection through the regulation of gene expression associated with antibacterial or antibody responses in Atlantic salmon (*Salmo salar*) [20] or zebrafish (*Danio rerio*) [21], respectively. In addition, comparative transcriptome analysis demonstrated a protective immune response through marked overexpression of humoral molecules in Arctic char (*Salvelinus alpinus*) administered with a vaccine against furunculosis [22]. However, little is known about the molecular mechanisms and signaling pathways that regulate the immune response following administration of a combination vaccine against *A. salmonicida* and *V. anguillarum* in rainbow trout.

Therefore, this study performed a transcriptome analysis to better understand the immune response in rainbow trout immunized with a combined vaccine of formalin-inactivated *A. salmonicida* and *V. anguillarum*. Gene expression profiles in the head kidney at days 1, 3, and 5 postvaccination were assessed by Q-PCR and RNA-seq transcript analysis.

## 2. Materials and Methods

### 2.1. Fish

Rainbow trout (*Oncorhynchus mykiss*) were purchased from a farm in Pyeong-Chang, Korea, and acclimated in pathogen-free conditions for a week before vaccination. Fish (average weight 33 ± 1.3 g) were kept in a continuously aerated 100-L square tank with running tap water after chlorine neutralization by addition of 2 ppm of sodium thiosulfate pentahydrate (Daejung) and maintained at 12 ± 1 °C [23]. Fish were treated according to GWNU’s Animal Care and Use Guidelines for Animal Welfare to minimize pain during the experiment.

### 2.2. Vaccine Preparation

Highly pathogenic *A. salmonicida* RTDH [23] and *V. anguillarum* RTBHR isolated from diseased rainbow trout from farms were used for vaccine production. In shaking incubators at 180 rpm, a single colony of *A. salmonicida* RTDH or *V. anguillarum* RTBHR isolates was incubated in tryptic soy broth (TSB) at 20 °C for 48 h or TSB supplemented with 2% sodium chloride at 25 °C for 24 h, respectively. Bacteria were inactivated in 2% formalin (Showa) for 3 h, centrifuged at 7000× *g* for 25 min, washed three times with sterile phosphate-buffered saline (PBS; Welgene, Gyeongsan, Korea), and adjusted to 200 mg/mL in sterile PBS. A combined vaccine (AV) was prepared by mixing equal amounts of formalin-killed *A. salmonicida* and *V. anguillarum* cells and stored at 4 °C.

### 2.3. Vaccination and Sampling

A total of 48 fish were divided into two groups and intraperitoneally injected with 100 μL of AV or sterile PBS as a negative control. At days 1, 3, and 5, eight fish per group at each time point were anesthetized with 2-phenoxyethanol and sacrificed by cutting the spinal cord. Head kidneys were collected aseptically, immediately frozen in liquid nitrogen, and stored at −70 °C until RNA isolation.

### 2.4. Q-PCR

Frozen head kidneys from each fish were crushed in liquid nitrogen in a mortar with a pestle, and total RNA was isolated using Qiazol (Qiagen, Hilden, Germany) according to the manufacturer’s instructions [24]. The extracted RNA (0.1–0.5 μg/μL) was immediately processed for reverse transcription using RevertAid H Minus Reverse Transcriptase and random primers according to the manufacturer’s instructions (Thermo Fisher Scientific). Briefly, 1 μL random primer (Thermo Fisher Scientific, Waltham, MA, USA) was added to 12 μL RNA and reacted at 65 °C for 5 min. For each sample, 4 μL of 5X Reaction Buffer Mix (Thermo), 0.5 µL of RiboLock RNase inhibitor (20 U/mL; Thermo), 2 μL of 10mM dinucleoside triphosphate (dNTP) mix (Thermo), and 0.5 μL of RevertAid H Minus Reverse Transcriptase (100 U/mL, Thermo) were added. The reaction mixture was incubated at 25 °C for 10 min, at 42 °C for 2 h, and at 70 °C for 10 min, and the final product was diluted by adding 380 μL of TE buffer.

Gene expression was analyzed by Q-PCR using the Quantstudio (Applied Biosystems, Foster City, CA, USA). Q-PCR was performed in a 20 μL reaction mixture containing 10 μL of SYBR Green Real-time PCR Master Mix (TaKaRa, Shiga, Japan), 0.4 mM each for forward and reverse primers, and 4 μL of cDNA using the following protocol: 10 min at 95 °C, template amplification for 45 cycles of denaturation for 10 s at 95 °C, annealing for primer specific temperature, and extension for 30 s at 72 °C. Q-PCR was performed in duplicates and transcription levels were calculated by absolute quantitative analysis using serially diluted references [25]. In addition, data normalization was performed using a housekeeping gene, i.e., elongation factor 1-alpha.

To verify the vaccine’s efficacy, the expression of adaptive immune genes, such as TCRα, T-bet, GATA3, mIgM, and sIgM, was analyzed. In addition, to validate the reliability of the RNA-seq data, nine differentially expressed genes (DEGs) were randomly selected and analyzed: heat shock protein family A (hsp70), C3, fibronectin 1, cluster of differentiation-22 (CD22), C-C motif chemokine ligand 19, hepcidin antimicrobial peptide 1 hamp-1, dnaJ heat shock protein family, and asteroid homolog 1. Primer sequences are listed in Supplementary Table S1.

### 2.5. Transcriptome Analysis

#### 2.5.1. RNA Library Construction and Mass Sequencing

RNA library construction and sequencing of two biological replicates for the PBS group and one for the AV group at days 1, 3, and 5 were performed according to the general procedure of the Illumina platform [26]. Total RNA quality and quantity were calculated using the Quant-IT RiboGreen Assay Kit (Invitrogen, Waltham, MA, USA). RNA integrity was assessed using the TapeStation RNA ScreenTape (#5067-5576; Agilent Technologies, Santa Clara, CA, USA). A total RNA library was prepared using the TruSeq Stranded mRNA Sample Prep Kit (Illumina, San Diego, CA, USA). The cDNA library was sequenced using Illumina NovaSeq 6000 (Illumina, San Diego, CA, USA). The reliability of a small sample of RNA-seq data was verified in eight replicates at each time point in a group by assessing the expression of nine randomly selected genes by Q-PCR, as mentioned previously.

#### 2.5.2. Transcriptome Annotation

RNA-seq data were processed to obtain clean data after removing reads containing adapters or poly-N as well as low-quality reads (base quality < 20). The quality of Fastq format files was evaluated using FastQC software and visually confirmed. The RNA-QC-chain was used to remove low-quality reads containing adapters and poly-N to generate clean reads from raw reads and mapped to the *Oncorhynchus mykiss* reference genome (Accession No. GCF_002163495.1) using Tophat v 2.0. Clear reads were mapped to the rainbow trout reference genome (Accession No. GCF_002163495.1), and then the number and percentage of uniquely mapped reads were calculated. The known and new copies were identified in the TopHat sort results using Cufflinks v 2.1.1 [27] with optional multi-read correction, frag-bias-correct, and default parameters. Expression levels were assessed by calculating the expected number of fragments per kilobase of transcript sequences per million base pairs (FPKM). The correlation between each sample was calculated through the expression value of FPKM.

#### 2.5.3. DEGs’ Analysis

DEG analysis was performed using Cuffdiff to compare changes between the vaccine and control groups, with multiple read correction and fragment bias correction options added for accurate analysis. Genes with FPKM ratios greater than log2FC with a *p*-value of <0.05 corrected for error throughout multiple tests were considered significantly up- or downregulated, respectively, and were defined as DEGs.

DEGs were evaluated for Gene ontology (GO) enrichment analysis and Kyoto Encyclopedia of Genetic and Genomic (KEGG) pathway analysis using DAVID software to clarify the biological functions. In addition, annotations were made using a human database, as information on rainbow trout was not available in the DAVID database.

Protein–protein interactions (PPIs) were analyzed using STRING (https://string-db.org/, accessed on 29 August 2021), and a network for the selected DEGs was drawn using Cytoscape 3.8.2 software. Network parameters such as degree, betweenness centrality, and closeness centrality were calculated using a network analyzer in Cytoscape. On days 1, 3, and 5, the number of immunity-related genes identified by KEGG analysis was 59, 21, and 21, respectively. Moreover, we performed functional evidence-based network association predictions for a confidence score of 0.25.

### 2.6. Statistics

Statistical analysis was performed using unpaired student’s t-test to detect differences in gene expression between the AV and control groups using SPSS version 25 for Windows (IBM Inc., Armonk, NY, USA).

## 3. Results

### 3.1. Effect of the Vaccine on Adaptive Immune Gene Expression

**Figure 1:**
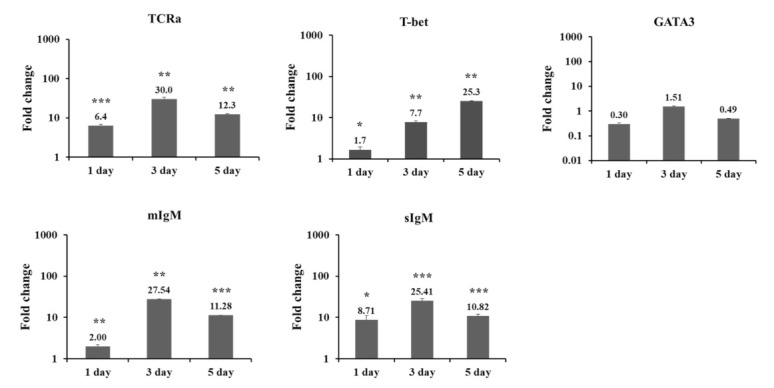
Q-PCR analysis of immune-related gene expression in the head kidney of rainbow trout injected with AV vaccine. Q-PCR data were normalized relative to the expression of the reference gene (EF-1α) and fold-change was calculated by dividing the ratio to EF-1α by control sample at days 1, 3, and 5. Data are presented as medians ± standard deviation (SD) (n = 8). Asterisks (*) indicate significant differences (* = *p* < 0.05, ** = *p* < 0.01, *** = *p* < 0.001) between the PBS and AV vaccine groups.

Genes responsible for adaptive immunity were analyzed by Q-PCR. TCRα gene expression was significantly upregulated in the vaccinated group at days 1, 3, and 5 (Figure 1). T-bet gene expression was significantly upregulated in a time-dependent manner. On days 1 and 5, GATA3 gene expression seemed to be downregulated, but not significantly. The mIgM and sIgM genes were significantly upregulated at days 1, 3, and 5.

### 3.2. Transcriptome Sequencing

After removing low-quality or duplicated reads and contaminated reads, clean reads with Q30 > 95% and Q20 > 98% were used for further analysis (Supplementary Table S2). Approximately 73,385,868 to 113,451,896 clean reads with a G/C content of approximately 48% were obtained. The unique mapping ratio of the nine transcriptome libraries of three AV- and six PBS-treated fish was more than 80% (Supplementary Table S3).

The correlation of gene expression levels between samples was >0.9, as verified by the square of the Pearson correlation coefficient (R^2^) (Supplementary Figure S1A). In pairwise comparisons, expression profiles between the vaccine and control groups were shown with a threshold of -log10 (*p*-value) ≥ 1.3 for downregulated and upregulated DEGs (Supplementary Figure S1B). Cluster analysis of DEGs indicated a difference in expression patterns between the vaccine and control groups (Supplementary Figure S1C).

### 3.3. Differential Expression Analysis

Differential expression analysis to compare the vaccinated and unvaccinated groups at days 1, 3, and 5 identified 862 (430 up- and 432 downregulated), 492 (204 up- and 288 downregulated), and 741 (270 up- and 471 downregulated) DEGs with statistical significance (log2(fold-change) >2 and *p*-value < 0.05), respectively (Supplementary Table S4).

### 3.4. GO Enrichment Analysis

**Figure 2:**
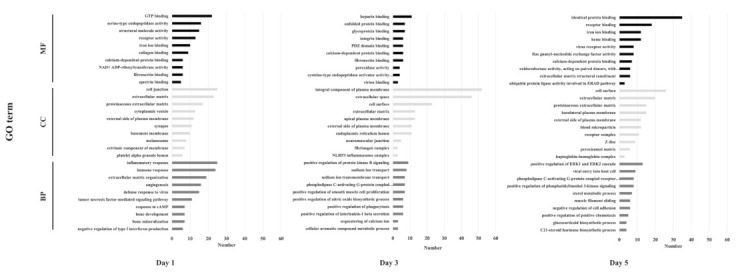
GO functional classification of DEGs between PBS and AV groups at days 1, 3, and 5. DEGs were annotated by biological process, cellular component, and molecular function. The X-axis is the number of annotated DEGs, and the Y-axis is the top 10 GO categories.

In GO annotations, DEGs were annotated into 377 GO (108 MF, 132 CC, 137 BP), 302 GO (60 MF, 180 CC, 62 BP), and 314 GO (115 MF, 129 CC, 70 BP) at days 1, 3 and 5, respectively (Figure 2). On day 1, MF categories were rich in “GTP binding”, “serine-type endopeptidase activity”, “structural molecule activity”, “receptor activity”, “iron ion binding”, and “collagen-binding”. CC categories were rich in “cell junction”, “extracellular matrix”, “proteinaceous extracellular matrix”, “cytoplasmic vesicle”, and “external side of plasma membrane, synapse”. BP categories were rich in “inflammatory response”, “immune response”, “extracellular matrix organization”, “angiogenesis”, “defense response to virus”, and “tumor necrosis factor-mediated signaling pathway”.

On day 3, MF categories were rich in “heparin-binding”, “unfolded protein binding”, and “glycoprotein binding”. CC categories were rich in “integral component of plasma membrane”, “extracellular space”, “cell surface”, “extracellular matrix”, “apical plasma membrane”, and “external side of plasma membrane”. BP categories were rich in “positive regulation of protein kinase B signaling”, “sodium ion transport”, “sodium ion transmembrane transport”, and “phospholipase C-activating G-protein coupled receptor signaling pathway”, and “positive regulation of smooth muscle cell proliferation” was abundant.

On day 5, MF categories were rich in “identical protein binding”, “receptor binding”, “iron ion binding”, and “heme binding”. CC categories were rich in “cell surface”, “extracellular matrix”, “proteinaceous extracellular matrix”, and “basolateral plasma membrane”. Finally, BP categories were rich in “positive regulation of extracellular signal-regulated kinase 1 (ERK1) and ERK2 cascade”, “viral entry into host cell”, “phospholipase C-activating G-protein coupled receptor signaling pathway”, and “positive regulation of phosphatidylinositol 3-kinase signaling”.

### 3.5. KEGG Pathway Enrichment Analysis

KEGG enrichment analysis identified 23, 13, and 22 pathways significantly enriched in DEG at days 1, 3, and 5, respectively (Table 1). On day 1, 177 annotated genes were grouped into 12 categories comprising 23 known KEGG pathways. The most significantly abundant KEGG pathways were “cytokine–cytokine receptor interaction”, “CAMs”, “neuroactive ligand–receptor interaction”, and “proteoglycans in cancer”. On day 3, 86 annotated genes were classified into 10 categories consisting of 13 KEGG pathways, including “proteoglycans in cancer”, “CAMs”, “protein processing in endoplasmic reticulum”, and “influenza A”. On day 5, 203 annotated genes were classified into 14 categories consisting of 22 KEGG pathways, and the significantly enriched pathways were “metabolic pathways”, “neuroactive ligand–receptor interaction”, “biosynthesis of antibiotics”, and “carbon metabolism”.

### 3.6. PPI Analysis of DEGs

**Figure 3:**
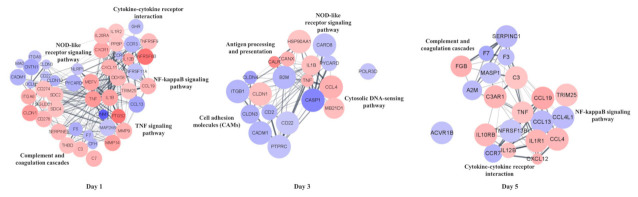
Protein–protein interaction (PPI) networks of immune-related DEGs at days 1, 3, and 5. Nodes represent genes, and links represent networks (genes can be linked by more than one type of network). Proteins are represented by nodes, and interactions are represented by edges. The number of edges is related to the strength of the interaction. The red and blue colors represent the upregulated and downregulated genes. The size of the node is inversely proportional to the *p*-value.

For the PPI network analysis of DEG, 59 genes at day 1, 21 genes at day 3, and 21 genes at day 5 identified in the KEGG analysis were used. In the PPI network analysis, a total of 486, 156, and 156 interactional relationships were identified between 59, 21, and 21 genes in the DEG at days 1, 3, and 5, respectively (Figure 3). The TNF gene showed the highest centrality in the centrality test at days 1, 3, and 5, followed by IL-1β at days 1 and 3, C3 at day 5 (Supplementary Table S5).

### 3.7. Analysis of DEGs in Immune-Related Pathways

A total of 75 (38 upregulated and 37 downregulated) annotated genes were identified to be involved in immune system-related KEGG pathways, including cytokine–cytokine receptor interaction, NF-kappaB signaling pathway, TNF signaling pathway, NOD-like receptor signaling pathway, cytoplasmic DNA sensing pathway, CAM, complement and coagulation cascade, and antigen processing and presentation (Table 2).

### 3.8. Validation of RNA-seq Results

**Figure 4:**
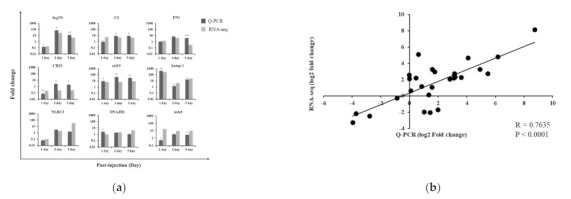
Validation of RNA-seq data. (**a**) Comparison of the fold-change of the expression of nine selected DEGs as determined by Illumina NovaSeq sequencing and Q-PCR. The DEGs were amplified in the head kidney of rainbow trout injected with AV vaccine. EF-1α was used as an internal reference gene. Data are presented as medians ± SD (n = 8). Asterisks (*) indicate significant differences (* = *p* < 0.05, ** = *p* < 0.01, *** = *p* < 0.001) between the PBS and AV vaccine groups. Hsp70, heat shock protein 70. C3, complement C3; FN1, fibronectin 1; CD22, cluster of differentiation 22; ccl19, C-C motif chemokine ligand 19; hamp-1, hepcidin antimicrobial peptide 1; NLRC3, NLR family CARD domain containing 3; DNAJB1, DnaJ homolog subfamily B member 1; aste1, asteroid homolog 1. (**b**) Correlation analysis of RNA-seq and Q-PCR in gene expression.

The reliability of RNA-seq data was validated as nine gene expression patterns were similar to Q-PCR data from eight fish samples per group at each time point (Figure 4a). Furthermore, the correlation between the transcriptome and Q-PCR data was 0.7635 in PBS vs. AV (Figure 4b).

## 4. Discussion

Numerous reports indicate that vaccines against *A. salmonicida* and *V. anguillarum* play an important role during bacterial infection in rainbow trout. In addition, it is known that vaccination using the injection route already shows an excellent protective effect [28]. Indeed, a correlation between protection and antibody titer has been demonstrated [28]. Furthermore, the various expression levels of T cells, transcription factors, or cytokines presented in infection studies of *A. salmonicida* and *V. anguillarum* provided basic information on the immune mechanism of the host’s response [29,30]. In addition, recent studies on transcriptome have provided an insight into the immune response during bacterial infection in fish. However, little is known about the pathways regulating the initial immune response administration after administering the combination vaccine against *A. salmonicida* and *V. anguillarum*. Therefore, in this study, RNA-seq transcript analysis was performed in the head kidney at days 1, 3, and 5 after vaccination to better understand the initial immune response of rainbow trout immunized with the mixed vaccine.

In this study, the AV vaccine was confirmed to have induced adaptive immunity in the head kidney, as TCRα, T-bet, mIgM, and sIgM genes were all upregulated in the vaccinated group. Upregulation of TCRα and T-bet indicates the activation of T cells, which recognize peptides presented by MHC via TCRs and initiate the adaptive immune response [31]. T-bet is a transcriptional regulator for Th-1 differentiation and interferon-γ production [32,33]. In this study, GATA3 gene expression seemed to be downregulated, but not significantly. Though GATA3 is a major transcription factor that regulates Th-2 differentiation [32,33], humoral immunity was also activated as the expression of mIgM and sIgM genes was significantly upregulated at days 1, 3, and 5. According to a previous study, T-bet and GATA3 genes were hardly expressed in the head kidney of rainbow trout infected with *A. salmonicida* at days 1, 2, and 5 [29]. Previously, recognition of inactivated *A. salmonicida* in rainbow trout has been shown to increase IgM-positive B cells [34].

**Figure 5:**
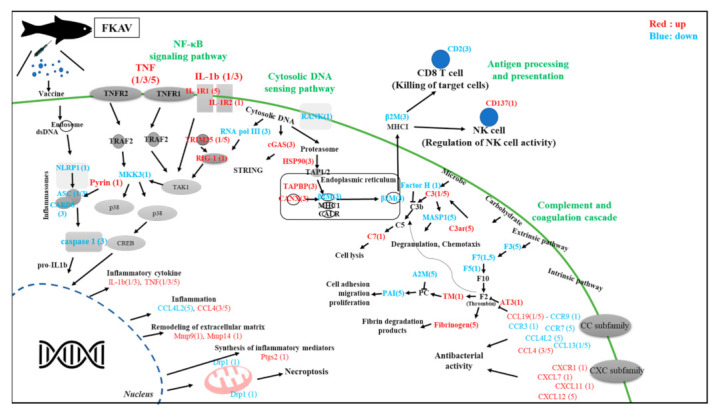
Presentation of putative immune pathways at days 1, 3, and 5 in AV group. DEGs regulated by AV vaccine are shown in red (upregulated) or blue (downregulated). The number next to the gene indicates the time point of reaction. Black arrows show activation and regulatory responses of downstream pathways.

In this study, the transcriptome of rainbow trout was characterized after the administration of the AV vaccine for the first time. The greatest changes in gene expression were found at day 1, and the most downregulated genes were found at day 5. In addition, eight immune system-related KEGG pathways, including cytokine–cytokine receptor interaction, NF-kappaB signaling pathway, TNF signaling pathway, NOD-like receptor signaling pathway, cytosolic DNA sensing pathway, CAM, complement and coagulation cascade, and antigen processing and presentation, were identified. These pathways are suggested to play an important role in the vaccine’s immune response of rainbow trout (Figure 5). Thus, it can be postulated that the vaccine was recognized by immune cells in the head kidney and initially activated cytokine–cytokine receptor interactions, subsequently enhancing transcription of immune genes and inflammatory factors through downstream pathways such as NF-kappaB and TNF signaling pathways. These are known to regulate many genes involved in various immune and inflammatory responses [35].

Inflammation acts as the first step in immune regulation against infection or stimuli [36]. In particular, proinflammatory cytokines, such as IL-1 and TNFα, act as important defense mechanisms against pathogens [36]. In our study, the combined vaccine upregulated IL-1β and TNFα gene expressions at days 1 and 3, or at days 1, 3, and 5. Recently, juvenile rainbow trout immunized with a multivalent vaccine combining *A. salmonicida*, *V. anguillarum* (O1, O2a), and *Y. ruckeri* (O1 biotypes 1 and 2) showed significant gene expression changes in the liver and spleen [17]. In particular, the spleen of vaccinated fish showed the most remarkable change in SAA produced by inflammatory cytokines IL-1b, IL-6, and TNFα after 8 weeks of vaccination [17]. In earlier studies, *V. anguillarum* infection was shown to result in a significant upregulation of IL-1β in teleosts, including Atlantic cod (*Gadus morhua*), gilthead seabream (*Sparus aurata*), and European sea bass (*Dicentrarchus labrax*) [37,38,39]. Furthermore, IL-1β and TNF act synergistically, with TNF being the first cytokine to follow IL-1β surge in the inflammatory response [40]. Therefore, administering rainbow trout with the mixed vaccine initially triggers the production of infectious cytokines such as TNF and IL-lβ and can produce acute proteins. However, although NF-κB is known to be involved in inflammasome regulation [41], inflammasome-related genes (NLRP1, ASC, and CARD8) were downregulated at days 1 and 3 in these experiments. The inflammasome is a cytoplasmic multiprotein that senses a variety of stimuli and regulates homeostasis [42]. NLRP1, ASC, and CARD8 form an inflammasome complex that is normally inactive until activated by pathogen-associated molecular patterns and damage-associated molecular patterns and induces innate immunity [43]. In mammals, this complex stimulates the activation of caspase-1, the most important inflammatory protease responsible for the formation and secretion of potent proinflammatory cytokines, such as IL-1β [44].

Caspases in mammals are classified into apoptosis initiators and inflammatory caspases according to their function [45]. In particular, casp1, casp4, casp5, casp11, and casp12 are representative inflammatory caspases [9]. Previous studies have identified a total of 18 caspase genes in the genome of rainbow trout and observed that the subfamily of inflammatory caspase contains only casp1a and casp1b [9]. In this study, caspase 1 gene expression was downregulated at day 3. Although sea bass and avian IL-1β are specifically cleaved by caspase-1 at phylogenetically conserved aspartates, distinct from the cleavage site of mammalian IL-1β [44], it is not clear whether downregulated caspase 1 gene expression affected IL-1β activity in this study. The precise function of caspase 1 in the processing of IL-1β in rainbow trout remains to be elucidated in more detail.

The internalized vaccine can be recognized by the cytosolic DNA sensing pathway as cyclic GMP–AMP synthase (cGAS) that was upregulated at day 3. The cGAS catalyzes cyclic GMP–AMP (cGAMP) synthesis, and cGAMP has been identified as the only universal cytoplasmic DNA sensor in various cell types that detects double-stranded DNA [46]. Recently, a cGAS-like gene was cloned in Grass carp (*Ctenopharyngodon idellus*) and found to be a negative modulator for IFN reactions by targeting the stimulator of interferon genes 1 (String1) [47]. The RPC6 gene, which recognizes cytosolic DNA, was downregulated on day 3. In addition, downstream pathway genes, tripartite motif-containing 25 (TRIM25), and retinoic acid-inducible gene (RIG)-I were upregulated at day 1/5 and day 1, respectively. RIG-I and TRIM25 are important initiators of the early immune response to viral infection [46]. RIG-I is a cytosolic pattern recognition receptor responsible for the type-1 interferon (IFN1) response [46]. RIG-I of zebrafish participates in the innate immune pathway similar to that of mammalian homologs [48]. It is also known that RNApol III can induce type I IFN through the RIG-I-like receptor signaling pathway [49], but RNApol III was downregulated at day 3 in this study. Thus, upregulation of cGAS and downregulation of RNA pol III at day 3 may lead to neutralization of IFN, which was activated by RIG-I and TRIM25 at day 1.

It can be assumed that the internalized antigens were processed for antigen presentation. In this study, hsp90, TAP-binding protein (TAPBP), calnexin (CANX), and beta-2-microglobulin (B2M) genes were upregulated and are known to be involved in signaling pathways for antigen processing and presentation. In a previous study, the spleen of rainbow trout stimulated with β-glucan was initially rich in antigen processing and presentation pathways, such as b2m, hsp90, and MHC1 due to *A. salmonicida* infection, and was involved in signaling T-cell receptors [8]. In addition, turbot administered with attenuated vaccines against *Vibrio anguillarum* and *Edwardsiella piscicida* increased adaptive immunity-related genes such as MHC I, MHC II, and TCR on day 1 after vaccination [18]. Antigens are processed and presented to specific lymphocytes after binding to MHC I or II [50]. Along with antigen presentation, CD137 gene expression was upregulated. This gene is expressed in activated T cells, dendritic cells, B cells, NK cells, neutrophils, and macrophages [51]. Crosslinking CD137 to CD137L on antigen-presenting cells increases T-cell proliferation, IL-2 secretion, survival, and cytolytic activity, indicating that it may also have triggered an antigen-specific immune response [51]. Therefore, the mixed vaccine may have prompted the head kidney of rainbow trout to first capture antigens and then deliver them to antigen-presenting cells.

In the chemokine pathway, four CXC subfamily members, CXCL7, CXCL11, CXCL12, and CXCR1, were upregulated, whereas six CC subfamily members showed variable expression patterns since CCL 4 and 19 were upregulated but CCL4L2, CCL 13, CCR7, and CCR9 were downregulated. CC and CXC chemokines have been identified in the teleost and have potential roles in homeostasis, signaling, inflammation, and immune activity [52,53]. In mammals, chemokines are among the more commonly used adjuvants for vaccination because they attract immune cells into the vaccinated site [54]. The coagulation cascade is key in maintaining hemostasis, and its activation can prevent blood loss in damaged vessels as well as repair vessels during inflammation [55]. In this study, F3 formed a complex with F7 and activated F10, but it was downregulated on day 1 and day 5. The primary function of this system is not to neutralize invading pathogens, but to maintain the integrity of the circulatory system in the event of injury [55].

The complement system promotes the ability to clear pathogens through opsonization, phagocytosis, and B cell activation [56,57]. It is an important part of innate immunity and is significantly upregulated in response to bacteria in several teleosts, including rainbow trout, catfish (*Silurus meridionalis*), and grass carp (*Ctenopharyngodon idella*) [58,59,60]. The C3 and C7 genes are conserved in rainbow trout and contain several complement components [61]. MBL-associated serine protease (MASP) is a proteolytic enzyme that activates the mammalian complement system through the lectin pathway [62]. Our study showed the C3 gene was upregulated by days 1 and 5, and biological relevance was also high in the PPI network analysis. Furthermore, the C7 gene was upregulated on day 1 and the MASP1 was downregulated on day 5. A previous study found that the gene expression of C3 was upregulated in the liver of rainbow trout immunized with a multivalent vaccine combining *A. salmonicida*, *V. anguillarum* (O1, O2a), and *Y. ruckeri* (O1 biotypes 1 and 2) [17]. The head kidney and spleen of rainbow trout with severe symptoms due to *V. anguillarum* infection showed a downregulated complement cascade and decreased defense against pathogen infection [63]. In addition, the expression of c1ql2, C3, C5, C7, C9, CFB, and complement factor H (CFH) was regulated in rainbow trout stimulated with β-glucan upon infection with *A. salmonicida*, suggesting improved immune enhancement [8]. Recently, Arctic Charr, a salmonid fish, showed marked significant overexpression of innate humoral molecules when a commercial furunculosis vaccine was administered [22].

## 5. Conclusions

This study suggests that the combination vaccine activated immune pathways that are important in the early immune responses of rainbow trout. In particular, activating the complement system, chemokines, and inflammatory cytokines activated immune responses through downstream signaling pathways. Upregulation of hsp90, TAPBP, and CANX genes induced the antigen processing and presentation pathway so that T cells can recognize the presence of a vaccine and trigger an immune response. Furthermore, the combined vaccine elicited a cell-mediated and humoral immune response, as evidenced by upregulated TCRα, T-bet, and IgM gene expression. This is the first report of a combination vaccine administered to rainbow trout to the best of our knowledge. This study enhances our understanding of immunity that occurs in response to a combined vaccine composed of *A. salmonicida* and *V. anguillarum,* and it lays the foundation for improving vaccine formulations in the future.

## Figures and Tables

**Table 1:** KEGG classification of differentially expressed genes (DEGs) between PBS and AV groups at days 1, 3, and 5.

Time (Day)	Category	Pathway ID	Pathway Terms	No. of DEGs	Fold Enrichment
Day 1	Signaling molecules and interaction	hsa04060	Cytokine–cytokine receptor interaction	16	2.35
	Signaling molecules and interaction	hsa04514	Cell adhesion molecules (CAMs)	15	3.77
	Signaling molecules and interaction	hsa04080	Neuroactive ligand–receptor interaction	14	1.80
	Cancer: overview	hsa05205	Proteoglycans in cancer	13	2.32
	Infectious disease: viral	hsa05164	Influenza A	10	2.05
	Signal transduction	hsa04064	NF-kappa B signaling pathway	8	3.28
	Digestive system	hsa04974	Protein digestion and absorption	8	3.24
	Infectious disease: viral	hsa05160	Hepatitis C	8	2.14
	Infectious disease: parasitic	hsa05144	Malaria	7	5.09
	Immune system	hsa04610	Complement and coagulation cascades	7	3.62
	Infectious disease: parasitic	hsa05146	Amoebiasis	7	2.35
	Signal transduction	hsa04668	TNF signaling pathway	7	2.33
	Infectious disease: parasitic	hsa05140	Leishmaniasis	6	3.01
	Digestive system	hsa04970	Salivary secretion	6	2.49
	Signaling molecules and interaction	hsa04512	ECM–receptor interaction	6	2.46
	Endocrine system	hsa04913	Ovarian steroidogenesis	5	3.64
	Carbohydrate metabolism	hsa00620	Pyruvate metabolism	5	4.46
	Amino acid metabolism	hsa00270	Cysteine and methionine metabolism	5	4.69
	Amino acid metabolism	hsa00260	Glycine, serine, and threonine metabolism	5	4.57
	Infectious disease: bacterial	hsa05134	Legionellosis	5	3.30
	Immune system	hsa04621	NOD-like receptor signaling pathway	5	3.18
	Lipid metabolism	hsa00590	Arachidonic acid metabolism	5	2.92
	Infectious disease: parasitic	hsa05143	African trypanosomiasis	4	4.32
Day 3	Cancer: overview	hsa05205	Proteoglycans in cancer	9	2.13
	Signaling molecules and interaction	hsa04514	Cell adhesion molecules (CAMs)	8	2.67
	Folding, sorting, and degradation	hsa04141	Protein processing in the endoplasmic reticulum	8	2.25
	Infectious disease: viral	hsa05164	Influenza A	8	2.18
	Signal transduction	hsa04020	Calcium signaling pathway	8	2.12
	Infectious disease: bacterial	hsa05133	Pertussis	6	3.80
	Immune system	hsa04621	NOD-like receptor signaling pathway	6	5.08
	Immune system	hsa04623	Cytosolic DNA sensing pathway	6	4.45
	Nucleotide metabolism	hsa00240	Pyrimidine metabolism	6	2.82
	Global and overview maps	hsa01200	Carbon metabolism	6	2.52
	Infectious disease: bacterial	hsa05134	Legionellosis	5	4.39
	Amino acid metabolism	hsa00260	Glycine, serine, and threonine metabolism	5	6.08
	Immune system	hsa04612	Antigen processing and presentation	5	3.12
Day 5	Global and overview maps	hsa01100	Metabolic pathways	50	1.43
	Signaling molecules and interaction	hsa04080	Neuroactive ligand–receptor interaction	14	1.76
	Metabolism of terpenoids and polyketides	hsa01130	Biosynthesis of antibiotics	13	2.14
	Global and overview maps	hsa01200	Carbon metabolism	12	3.71
	Signaling molecules and interaction	hsa04060	Cytokine–cytokine receptor interaction	12	1.72
	Lipid metabolism	hsa00140	Steroid hormone biosynthesis	9	5.42
	Signal transduction	hsa04064	NF-kappa B signaling pathway	8	3.21
	Immune system	hsa04610	Complement and coagulation cascades	8	4.05
	Digestive system	hsa04976	Bile secretion	8	4.05
	Carbohydrate metabolism	hsa00010	Glycolysis/Gluconeogenesis	8	4.17
	Endocrine system	hsa04922	Glucagon signaling pathway	7	2.47
	Infectious disease: parasitic	hsa05143	African trypanosomiasis	6	6.35
	Infectious disease: bacterial	hsa05134	Legionellosis	6	3.88
	Endocrine system	hsa04913	Ovarian steroidogenesis	6	4.28
	Global and overview maps	hsa01230	Biosynthesis of amino acids	6	2.91
	Xenobiotics biodegradation and metabolism	hsa00980	Metabolism of xenobiotics by cytochrome P450	6	2.83
	Metabolism of cofactors and vitamins	hsa00760	Nicotinate and nicotinamide metabolism	4	4.82
	Carbohydrate metabolism	hsa00630	Glyoxylate and dicarboxylate metabolism	4	5.17
	Amino acid metabolism	hsa00340	Histidine metabolism	4	6.35
	Carbohydrate metabolism	hsa00040	Pentose and glucuronate interconversions	4	4.23
	Amino acid metabolism	hsa00250	Alanine, aspartate, and glutamate metabolism	4	3.99
	Amino acid metabolism	hsa00260	Glycine, serine, and threonine metabolism	4	3.58

**Table 2:** Summary of DEGs in immune-related KEGG pathways.

Category/Gene Name	Description	Fold-Change/*p*-Value					
		Day 1			Day 3	Day 5	
**Cytokine–cytokine receptor interaction**							
TNF	Tumor necrosis factor	3.87	0.0016			2.11	0.00485
TNFRSF11A	TNF receptor superfamily member 11A	−2.18	0.00005				
TNFRSF13B	TNF receptor superfamily member 13B					−2.04	0.00065
TNFRSF6B	TNF receptor superfamily member 6B	5.39	0.00005				
TNFRSF9	TNF receptor superfamily member 9	2.43	0.00005				
IL1β	Interleukin 1 beta	3.46	0.00015				
IL10Rβ	Interleukin 10 receptor subunit beta					2.63	0.00005
IL12β	Interleukin 12B	3.33	0.0258			2.26	0.0314
IL1R1	Interleukin 1 receptor type 1					2.73	0.00005
IL1R2	Interleukin 1 receptor type 2	2.02	0.00005				
IL20RA	Interleukin 20 receptor subunit alpha	2.51	0.00005				
CCL13	C-C motif chemokine ligand 13	−3.4	0.00005			−2.5	0.00005
CCL19	C-C motif chemokine ligand 19	2.74	0.00005			3.28	0.00005
CCL4	C-C motif chemokine ligand 4					2.64	0.00005
CCL4L2	C-C motif chemokine ligand 4 like 2					−2.43	0.00015
CCR3	C-C motif chemokine receptor 3	−2.68	0.00005				
CCR7	C-C motif chemokine receptor 7					−2.85	0.0189
CCR9	C-C motif chemokine receptor 9	−2.48	0.046				
CXCL11	C-X-C motif chemokine 11	2.78	0.00115				
CXCL12	C-X-C motif chemokine ligand 12					2.83	0.0495
CXCR1	C-X-C motif chemokine receptor 1	3.29	0.0005				
ACVR1B	Activin A receptor type 1B					−2.23	0.0009
GHR	Growth hormone receptor	−2.25	0.0072				
PPBP	Pro-platelet basic protein	2.16	0.00855				
**NF-kappaB signaling pathway**							
TNF	Tumor necrosis factor	3.87	0.0016			2.11	0.00485
TNFRSF11A (RANK)	TNF receptor superfamily member 11A	−2.18	0.00005				
IL1β	Interleukin 1 beta	3.46	0.00015				
IL1R1	Interleukin 1 receptor type 1					2.73	0.00005
CXCL12	C-X-C motif chemokine ligand 12					2.83	0.0495
CCL4L2	C-C motif chemokine ligand 4 like 2					−2.43	0.00015
CCL4	C-C motif chemokine ligand 4					2.64	0.00005
CCL19	C-C motif chemokine ligand 19	2.74	0.00005			3.28	0.00005
CCL13	C-C motif chemokine ligand 13	−3.4	0.00005			−2.5	0.00005
TRIM25	Tripartite motif-containing 25	2.09	0.00005			2.15	0.00015
PTGS2	Prostaglandin-endoperoxide synthase 2	5.34	0.01175				
DDX58 (RIG-I)	DExD/H-Box Helicase 58	2.62	0.00005				
**TNF signaling pathway**							
TNF	Tumor necrosis factor	3.87	0.0016				
IL1β	Interleukin 1 beta	3.46	0.00015				
PTGS2	Prostaglandin-endoperoxide synthase 2	5.34	0.01175				
MMP9	Matrix metalloproteinase-9	2.81	0.00005				
MMP14	Matrix metallopeptidase 14	3.65	0.00005				
MAP2K6 (MKK6)	Mitogen-activated protein kinase kinase 6	−2.15	0.00005				
DNM1L (Drp1)	Dynamin 1 Like	−6.23	0.03775				
**NOD-like receptor signaling pathway**							
TNF	Tumor necrosis factor	3.87	0.0016	2.82	0.03365		
IL1β	Interleukin 1 beta	3.46	0.00015	2.34	0.01225		
PYCARD (ASC)	Apoptosis-associated speck-like protein containing a CARD	−2.89	0.00005	−2.18	0.02475		
NLRP1	NLR family pyrin domain containing 1	−2.16	0.0259				
MEFV (Pyrin)	MEFV innate immunity regulator, pyrin	3.43	0.00005				
HSP90AA1 (Hsp90)	Heat shock protein 90 alpha family class A member 1			2.06	0.00005		
CARD8	Caspase recruitment domain family member 8			−2.25	0.00005		
**Cytosolic DNA sensing pathway**							
IL1β	Interleukin 1 beta			2.34	0.01225		
CCL4	C-C motif chemokine ligand 4			2.92	0.00005		
CASP1	Caspase 1			−4.55	0.00035		
PYCARD (ASC)	Apoptosis-associated speck-like protein containing a CARD			−2.18	0.02475		
POLR3D (RNA pol III)	RNA polymerase III Subunit D			−2.08	0.02185		
MB21D1(cGAS)	Cyclic GMP–AMP Synthase			2.35	0.0078		
**Cell adhesion molecules (CAMs)**							
CD2	Cluster of differentiation 2			−2.81	0.024		
CD22	Cluster of differentiation 22	−2.18	0.03475	−2.02	0.001		
CD274	Cluster of differentiation 274	2.32	0.00005				
CD276	Cluster of differentiation 276	2.69	0.0001				
CLDN1	Claudin 1	3.78	0.00005	2.25	0.0005		
CLDN10	Claudin 10	−2.96	0.03065				
CLDN3	Claudin-3	−2.24	0.03815	−2.87	0.0136		
CLDN4	Claudin 4			−3.49	0.0321		
CNTN1	Contactin 1	−3.33	0.00125				
CADM1	Cell adhesion molecule 1	−2.81	0.00005	−2.66	0.00005		
ITGA6	Integrin subunit alpha 6	2.97	0.00565				
ITGA8	Integrin subunit alpha 8	−2.28	0.01655				
ITGB1	Integrin beta-1			−2.73	0.00005		
MAG	Myelin associated glycoprotein	−2.09	0.0414				
OCLN	Occludin	−2.57	0.0476				
PTPRC	Protein tyrosine phosphatase receptor type C			−2.16	0.00005		
SDC2	Syndecan 2	2.4	0.00005				
SDC4	Syndecan 4	2.21	0.00005				
SIGLEC1	Sialic acid-binding Ig like lectin 1	2.41	0.04695				
**Complement and coagulation cascades**							
C3	Complement C3	2.57	0.0036			2.06	0.00005
C3AR1	Complement C3a receptor 1					2.74	0.00005
C7	Complement C7	2.85	0.00005				
CFH (Factor H)	Complement factor H	−2.75	0.0349				
MASP1	MBL-associated serine protease 1					−2.01	0.00735
A2M	Alpha-2-macroglobulin					−2.48	0.01375
F3	Coagulation factor III					−2.35	0.0262
F5	Coagulation factor V	−3.05	0.0147				
F7	Coagulation factor VII	−2.63	0.0329			−3.2	0.03335
FGB (Fibrinogen)	Fibrinogen beta chain					2.91	0.0063
SERPINC1 (AT3)	Serpin family C member 1					−2.56	0.0021
SERPINE1 (PAI)	Serine protease inhibitor (serpin) protein	2.63	0.00005				
THBD (TM)	Thrombomodulin	2.25	0.00005				
**Antigen processing and presentation**							
TNF	Tumor necrosis factor			2.82	0.03365		
B2M	Beta-2-microglobulin			−2.73	0.00045		
CALR	Calreticulin			4.36	0.037		
CANX	Calnexin			2.75	0.0272		
HSP90AA1 (Hsp90)	Heat shock protein 90 alpha family class A member 1			2.06	0.00005		

## Data Availability

The data that support the findings of this study are available from the corresponding author upon reasonable request.

## Supplementary Materials

The following are available online at https://www.mdpi.com/article/10.3390/vaccines9111234/s1. Figure S1: Quality assessment and comparisons of transcriptome data quality between PBS and AV groups. Table S1: Primers used for Q-PCR. Table S2: Summary statistics for the sequencing data on nine samples. Table S3: Statistics of clean reads mapped onto reference genome. Table S4: Comparative analysis of DEG between PBS and AV. Table S5: Top 10 nodes for PPI network centrality analysis of immune-related DEGs.
