# Supplementary material for: Transcriptome Analysis in the Head Kidney of Rainbow Trout (Oncorhynchus mykiss) Immunized with a Combined Vaccine of Formalin-Inactivated Aeromonas salmonicida and Vibrio anguillarum
Source: Vaccines (Basel). 2021 Oct 22;9(11):1234. doi: 10.3390/vaccines9111234 (PMC8619301; doi:10.3390/vaccines9111234)
Supplement: Supplementary file 1 [file vaccines-09-01234-s001.zip › vaccines-1392953-supplementary.pdf]

Table S1. Primers used for Q-PCR.

Primer	Length	Tm (℃)	GC (%)	Size (bp)	Sequence (5`-3`)	Reference
T-bet_F	20	56.4	45	124	TTCTGCCATTTTGTGTCAGG	[31]
T-bet_R	21	59.4	48		TTCTCCATCCTATTGCTCCAG	
GATA3_F	20	58.4	50	161	TCCTGGAGAGAGGGATGAAA	
GATA3_R	20	60.5	55		AGCCCGAGACCTATAGCACA	
TCRα_F	25	60.9	36	122	ACGCACTTGGAATTATTCAACAAGA	In this study
TCRα_R	25	64.2	44		GCTTCACATTTCTCTGAACCACCTA	
mIgM_F	19	57.3	52.6	102	ATGGAAACCGACAGGGACA	
mIgM_R	19	57.3	52.6		CACCTTGATGGCAGTTGCT	
sIgM_F	19	58.4	50	110	GAACCTCAAACCAACCCAAC	
sIgM_R	20	58.4	50		GTAACACGCAGCAACACACA	
Hsp70_F	20	58.4	50	187	GTGGAGGAGTTCAAGAGGAA	
Hsp70_R	20	62.5	60		CCTCAAAACGAGCCCTGGTG	
C3_F	19	61.7	63	164	GATGGGGACGTCTGTGTCC	
C3_R	20	60.5	55		GGGAACTGACTACGAGACAC	
FN1_F	20	58.4	50	108	TGACAGACCATGGGTAAGGA	
FN1_R	20	58.4	50		GAATGCCTTCGACCTGAACT	
CD22_F	23	66.4	57	210	GGCCCAAAGAACACCTCAGTGTC	
CD22_R	22	62.1	50		GGGCCTCACAGTAATATTCTCC	
ccl19_F	20	60.5	55	173	TTGCTGTCTCCTCACCACTG	
ccl19_R	20	60.5	55		TGTAGTCTGCCACCCACTTG	
hamp-1_F	20	58.4	50	171	AGTCCAGTTGGGGAACATCA	
hamp-1_R	20	60.5	55		GGTTTTAGTGCAGGCAGGTC	
NLRC3_F	20	60.5	55	171	GAACGAGGCAGAGAAGGATG	
NLRC3_R	20	58.4	50		GCCAGCTTTGCCAGTTTAAG	
DNAJB1_F	20	58.4	50	188	CGTGGTGTTTGTGGTCAAAG	
DNAJB1_R	20	58.4	50		TACGCTTCTTCATCCCAGGT	
aste1_F	20	60.5	55	175	TGGTGTGTCCCAGTCCACTA	
aste1_R	20	60.5	55		CCCTCTTTGGCTCTTCCTCT	

Table S2. Summary statistics for the sequencing data on 9 samples.

Time (day)	Sample[1]	Total Bases[2] (bp)	Raw read[3]	Clean read[4]	GC[5] (%)	AT[6] (%)	Q20[7] (%)	Q30[8] (%)
**1**	PBS 1	11,755,507,362	116,391,162	113,451,896 (97.6%)	48.80	51.20	98.44	95.44
	PBS 2	7,623,335,368	75,478,568	73,385,868 (97.4%)	49.58	50.42	98.30	95.12
	AV	10,267,691,512	101,660,312	99,256,038 (97.80%)	49.44	50.56	98.49	95.51
**3**	PBS 1	7,837,444,662	77,598,462	75,469,696 (97.5%)	49.09	50.91	98.36	95.32
	PBS 2	8,154,824,032	80,740,832	78,619,084 (97.5%)	48.88	51.12	98.38	95.33
	AV	10,043,467,270	99,440,270	96,791,774 (97.50%)	49.41	50.59	98.38	95.31
**5**	PBS 1	8,916,525,228	88,282,428	86,223,008 (97.8%)	49.47	50.53	98.56	95.78

PBS 2	9,910,185,650	98,120,650	95,487,188 (97.5%)	49.0	51.0	98.37	95.30
AV	9,145,109,236	90,545,636	88,224,742 (97.60%)	48.91	51.09	98.42	95.41

[1] PBS and AV represent the samples from the PBS or vaccine injection groups, respectively. [2] Total numbers of bases sequenced. [3] Total read counts of raw data. For Illumina paired-end sequencing, this value refers to the sum of read 1 and read 2. [4] Total read count after cleaning by FastQC. [5] GC content ratio of clean reads. [6] AT content ratio of clean reads. [7] Ratio of bases with phred quality score >20. [8] Ratio of bases with phred quality score >30.

Table S3. Statistics of clean reads mapped onto reference genome.

Time (day)	Sample	Number of Mapped Reads	Percentage of Mapped Reads (%)	Number of Uniquely Mapped Reads	Percentage of Uniquely Mapped Reads (%)
1	PBS 1	98,986,537	87.2	90,744,204	80.0
	PBS 2	64,278,162	87.6	59,266,630	80.8
	AV	87,578,166	88.2	80,182,679	80.8
3	PBS 1	66,021,957	87.5	60,649,197	80.4
	PBS 2	68,139,289	86.7	62,864,439	80.0
	AV	84,077,029	86.9	76,840,919	79.4
5	PBS 1	75,523,041	87.6	69,638,263	80.8
	PBS 2	83,154,279	87.1	76,600,416	80.2
	AV	76,916,458	87.2	70,654,311	80.1

Table S4. Comparative analysis of DEG between PBS and AV. a: Total number of DEGs in PBS and AV, b: AV is upregulated compared to PBS, c: AV is downregulated compared to PBS.

Time (day)	Group 1	Group 2	Number of genes		
			Total [a]	Up [b]	Down [c]
1			862	430	432
3	Control	AV	492	204	288
5			741	270	471

Table S5. Top 10 nodes for PPI network centrality analysis of immune-related DEGs. At days 1 (A), 3 (B) and 5 (C), 59, 21, and 21 genes are selected for network display with weight score of >0.25 in a total of 8 immune-related KEGG pathways.

Time (day)	Node name	Closeness Centrality	Betweenness Centrality	Degree
1	TNF	0.8704	0.3147	40
	IL1B	0.8246	0.2062	37
	MMP9	0.6912	0.0909	27
	OCLN	0.5465	0.0435	10
	C3	0.5663	0.0381	15
	MMP14	0.5529	0.0310	11
	MAG	0.5054	0.0278	5
	IL12β	0.4947	0.0251	5
	SERPINE1	0.5875	0.0218	15
	CFH	0.5281	0.0209	9
3	TNF	1.0000	0.2212	18
	IL1B	1.0000	0.2212	18
	PTPRC	0.7500	0.0376	12
	B2M	0.7500	0.0324	12

	CASP1	0.7200	0.0322	11
	ITGB1	0.6923	0.0307	10
	CALR	0.6429	0.0088	8
	PYCARD	0.6207	0.0075	7
	CD2	0.6429	0.0050	8
	HSP90AA1	0.6429	0.0046	8
**5**	TNF	0.9048	0.3086	17
	C3	0.7308	0.0849	12
	F3	0.7037	0.0657	11
	CCL4	0.7308	0.0562	12
	CXCL12	0.7037	0.0430	11
	CCL19	0.6552	0.0371	10
	C3AR1	0.6129	0.0296	7
	A2M	0.6333	0.0242	8
	CCR7	0.6333	0.0172	9
	IL1R1	0.6552	0.0149	9

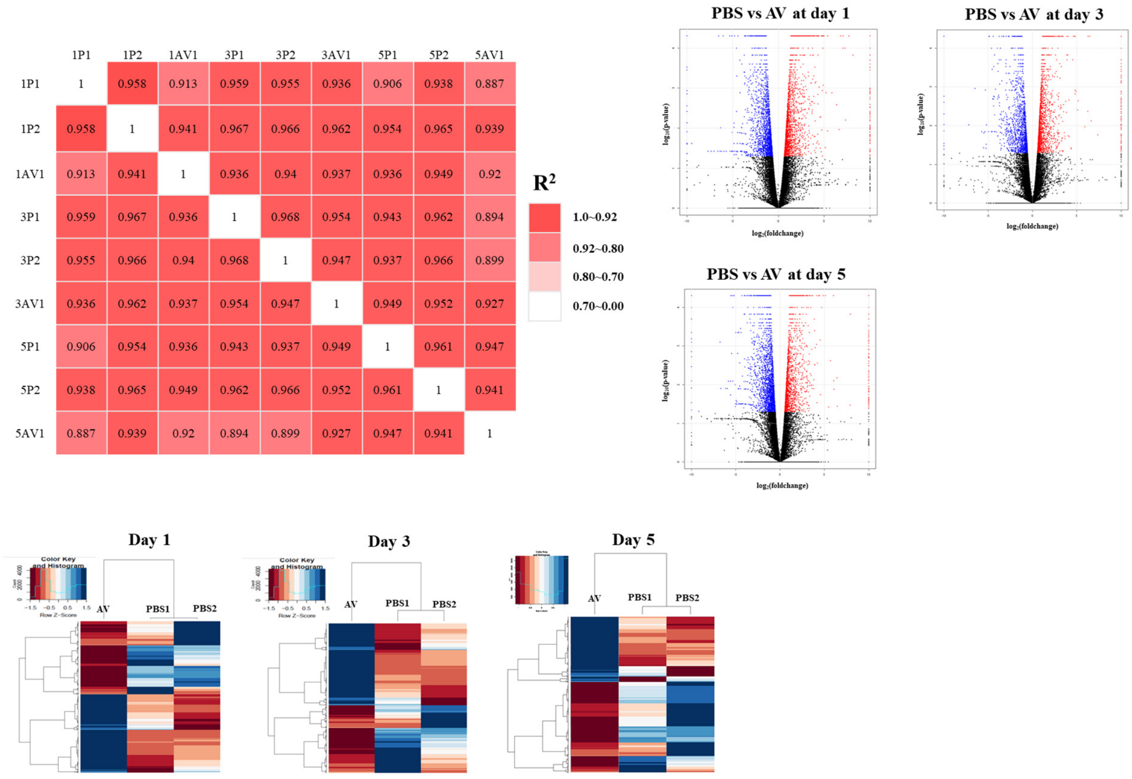

Figure S1. Quality assessment and comparisons of transcriptome data quality between PBS and AV groups. A) Correlation matrix of the transcriptome data of all samples. B) Volcano plot of differentially expressed genes (DEGs) distribution in the PBS vs AV pairwise comparisons. (C) Hierarchical cluster analysis of gene expression profiles in the PBS vs AV.
